# Beta and Gamma Human Herpesviruses: Agonistic and Antagonistic Interactions with the Host Immune System

**DOI:** 10.3389/fmicb.2017.02521

**Published:** 2018-01-05

**Authors:** Mario E. Cruz-Muñoz, Ezequiel M. Fuentes-Pananá

**Affiliations:** ^1^Laboratorio de Inmunología Molecular, Facultad de Medicina, Universidad Autónoma del Estado de Morelos, Cuernavaca, Mexico; ^2^Unidad de Investigación en Virología y Cáncer, Hospital Infantil de México Federico Gómez, Mexico City, Mexico

**Keywords:** herpesvirus, EBV, mutualistic relationship, inflammation, immunodeficiency, immunosuppression, autoimmunity, lymphoproliferation

## Abstract

Viruses are the most abundant and diverse biological entities in the planet. Historically, our main interest in viruses has focused on their pathogenic role, recognized by pandemics that have decimated the world population. However, viral infections have also played a major role in the evolution of cellular organisms, both through interchanging of genes with novel functions and shaping the immune system. Examples abound of infections that seriously compromise the host integrity, but evidence of plant and insect viruses mutualistic relationships have recently surfaced in which infected hosts are better suited for survival, arguing that virus-host interactions are initially parasitic but become mutualistic over years of co-evolution. A similar mutual help scenario has emerged with commensal gut bacteria. EBV is a herpesvirus that shares more than a hundred million years of co-evolution with humans, today successfully infecting close to 100% of the adult world population. Infection is usually acquired early in childhood persisting for the host lifetime mostly without apparent clinical symptoms. Disturbance of this homeostasis is rare and results in several diseases, of which the best understood are infectious mononucleosis and several EBV-associated cancers. Less understood are recently found inborn errors of the immune system that result in primary immunodeficiencies with an increased predisposition almost exclusive to EBV-associated diseases. Puzzling to these scenarios of broken homeostasis is the co-existence of immunosuppression, inflammation, autoimmunity and cancer. Homologous to EBV, HCMV, HHV-6 and HHV-7 are herpesviruses that also latently infect most individuals. Several lines of evidence support a mutualistic equilibrium between HCMV/EBV and hosts, that when altered trigger diseases in which the immune system plays a critical role. Interestingly, these beta and gamma herpesviruses persistently infect all immune lineages and early precursor cells. In this review, we will discuss the evidence of the benefits that infection of immune cells with these herpesviruses brings to the host. Also, the circumstances in which this positive relationship is broken, predisposing the host to diseases characterized by an abnormal function of the host immune system.

## Introduction

**Figure 1:**
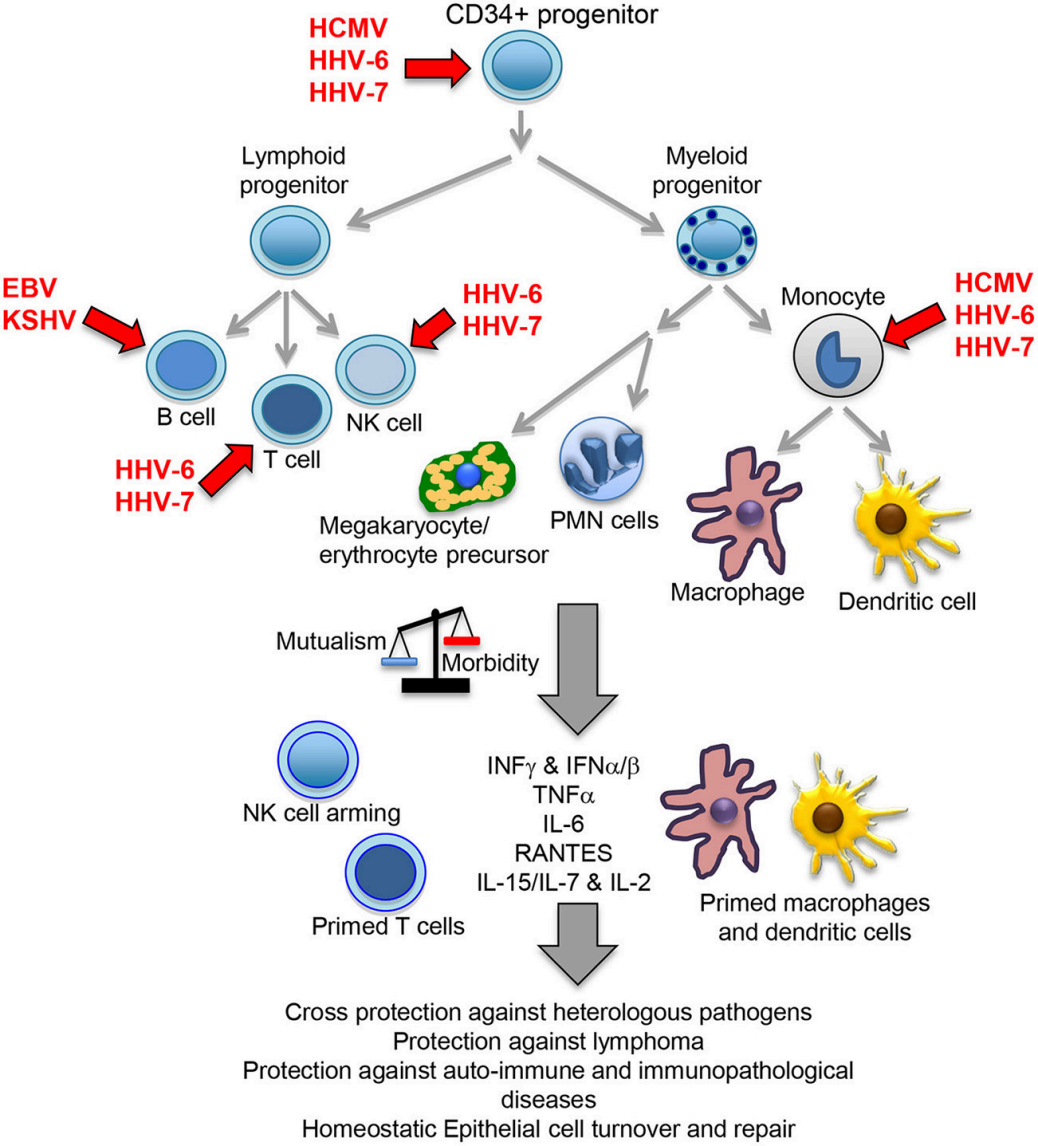
Beta- and gamma-herpesvirus mutualistic relationship with human host. While a restricted B cell tropism is usually found *in vivo* for gamma-herpesviruses, a wide tropism is observed for beta-herpesviruses that includes CD34 positive early progenitors, T cells, NK cells, monocytes, macrophages and dendritic cells. In homeostatic conditions, the herpesvirus immunomodulatory mechanisms positively influence host immunity, cross-protecting against heterologous pathogens through NK cell arming, and perhaps also due to their capacity to increase the numbers of T and NK cells, plus bystander activation through increased levels of local and systemic cytokines. Viral immunomodulation also improves tumor immunosurveillance, protects against auto-immune/immunopathological diseases and cooperate with other homeostatic processes, such as epithelial cell turnover and repair. A *primed* state indicates a possible pre-activated form of the immune cells triggered by IFNγ or other cytokines. PMN, polymorphonuclear cells.

The herpesvirus family consists of large enveloped viruses with a double stranded (ds) DNA genome. There are more than 100 different members of which 9 infect humans: herpes simplex type 1 and 2 (HHV-1 and HHV-2), varicella zoster virus (VZV/HHV-3), Epstein-Barr virus (EBV/HHV-4), cytomegalovirus (HCMV/HHV-5), HHV-6A, HHV-6B, HHV-7 and Kaposi sarcoma virus (KSHV/HHV-8). Herpesviruses are characterized by a bipartite life cycle alternating latent and lytic stages. While the lytic cycle should normally occur in mucosae aiming to transmit the virus to new hosts, latency permits establishing long-lasting chronic infections. Alpha herpesviruses (HHV-1, HHV-2 and HHV-3) are neurotropic viruses that establish latent persistent infections in nerve cells of the sensory ganglia. Beta- (HCMV, HHV-6A, HHV-6B, and HHV-7) and gamma-herpesviruses (EBV and KSHV) establish latent infection in cells of the immune system, persisting in CD34 positive progenitor cells and more differentiated lymphoid and myeloid immune cells (Figure 1). Because of this reason, these viruses are the focus of this review, and from now on the term herpesvirus will only refer to the beta and gamma members of the family. Similarly, HHV-6 will refer to both genetically distinct but structurally and functionally highly similar HHV-6A and HHV-6B. HHV-6 and HHV-7 are also known as the roseolovirus, because primary infection with these viruses causes roseola infantum, an illness characterized by rash and fever in children under the age of four.

Herpesvirus infections are among the most prevalent in the human population. For instance, 90% of 6 years old children are already infected with the roseolovirus in the US. Indeed, with the exception of KSHV, which is endemic only in certain Mediterranean and sub-Saharan African countries, most of the adult world population is infected with the other herpesviruses, particularly in developing countries. The prevalence of HCMV and EBV is about 70 and 95% in adults worldwide, with infection usually occurring during childhood and lasting for the host entire life. It is noteworthy that in spite of the billions of people infected a relatively few develop associated diseases, exposing a fine-tuned balance between virus and host in which both are preserved. The following diseases are associated with herpesvirus infection. EBV is accountable for several B cell lymphomas in immune-competent and immune-suppressed individuals, but also with (natural killer) NK and T cell lymphomas, carcinomas of the nasopharynx and stomach, and also with non-cancerous diseases that however increase the risk to develop lymphoma, such as infectious mononucleosis (IM), chronic active EBV (CAEBV) and hemophagocytic lymphohistiocytosis (HLH). KSHV is also associated with several neoplasias: Kaposi sarcoma (KS), multicentric castleman disease (MCD), and primary effusion lymphoma (PEL), and also with the KSV-inflammatory cytokine syndrome (KICS). HCMV primary infection or severe reactivation is associated with disease in the organs harboring the virus, mainly the liver, kidney, gastrointestinal track, lung, retina and brain. Primary infection during pregnancy can cause neurosensory damage to the unborn child, leading to hearing loss and mental retardation. An HCMV oncomudalator role has also been proposed in high-grade glioblastomas. HHV-6- and HHV-7-associated roseola infantum in rare cases can lead to seizure and encephalitis.

## The virobiome and evidence of mutualistic interactions

The positive role of the microbiome has been known for decades, particularly the role of gut bacteria to provide with nutrients, vitamins, digestive enzymes, and protection against invading pathogens. Although, it was first though that protection was based on a mere niche competition between normal resident and invading pathogen bacteria, today, it is clear that gut bacteria perform critical roles in the development and function of gut cell immune cells, innate and adaptive. Indeed, axenic mice develop hypoplastic gut lymphoid tissues. Moreover, these mice support a far-reaching role of the microbiome beyond the gut immune tissue, with systemic manifestations of abnormal function of the immune, cardiovascular and nervous systems (Carding et al., 2015; Ghaisas et al., 2016; Sherwin et al., 2016).

Viruses are one of the most primitive and the most abundant form of life on the planet. The virome is a component of the microbiome for which we are oblivious about its contribution to the host's wellness. While a mutualistic role seems counter-intuitive since viruses are obliged cellular parasites, they are an integral part of our evolution as evidenced by the fact that about 42% of our genetic makeup is constituted by viral sequences, illustrating the extensive host-virus exchange of genetic information (Lander et al., 2001; Katzourakis and Gifford, 2010; Parker, 2016). Most host-virus interactions probably start as parasitic and become mutualistic after thousand/millions of years of co-evolution. Although, our current knowledge is shaped almost exclusively by examples of infections compromising the integrity of the host, evidence of plant and insect viruses mutualistic alliances have recently surfaced in which the infected host is better suited for survival, similar to the mutual help scenario between commensal gut bacteria and humans. Plants with persistent viral infections exhibit an enhanced capacity to counteract environmental stress. For instance, infected plants are more resistant to plagues, drought or extreme temperatures. Viruses induce the synthesis of volatile substances that keep away parasitic insects while attracting the pollinating ones (Roossinck, 2015a). White clovers persistently infected with a cryptovirus are more efficient to form nitrogen-fixing root nodules in response to nitrogen availability (Nakatsukasa-Akune et al., 2005). Examples in insects also abound, infection by densovirus increases the life span and fecundity of bollworms and infected bollworms are also more resistant to biopesticides (Xu et al., 2014).

There are also millions (estimated between 100 and 400) of years of co-evolution between herpesviruses and humans. The rate of infected to diseased people and the length of the virus-host interactions strongly support that herpesviruses are better classified as human virobionts and part of our normal microbiome than to true pathogens. This lasting relationship is characterized by the establishment of latency stages in which the viral genome persists in the nucleus of the infected cell, generally as extra-chromosomal episomes, with HHV-6 as the only exception with about 1% of the population carrying the virus integrated in telomeric regions. Latency is characterized by a restricted pattern of viral gene expression, e.g. EBV expresses up to nine proteins and several non-coding RNAs, while it expresses up to 100 genes during the lytic cycle. Moreover, latent gene expression is dynamic and infected memory B cells often lack expression of any EBV viral protein. Latent expression is enriched with viral genes with the capacity to subvert important cellular processes, such as differentiation, survival and proliferation, while lytic expression includes genes with transcriptional and polymerase activities and structural proteins, aiming to produce new infectious viruses.

## The immunomodulatory capacity of herpesviruses

**Figure 2:**
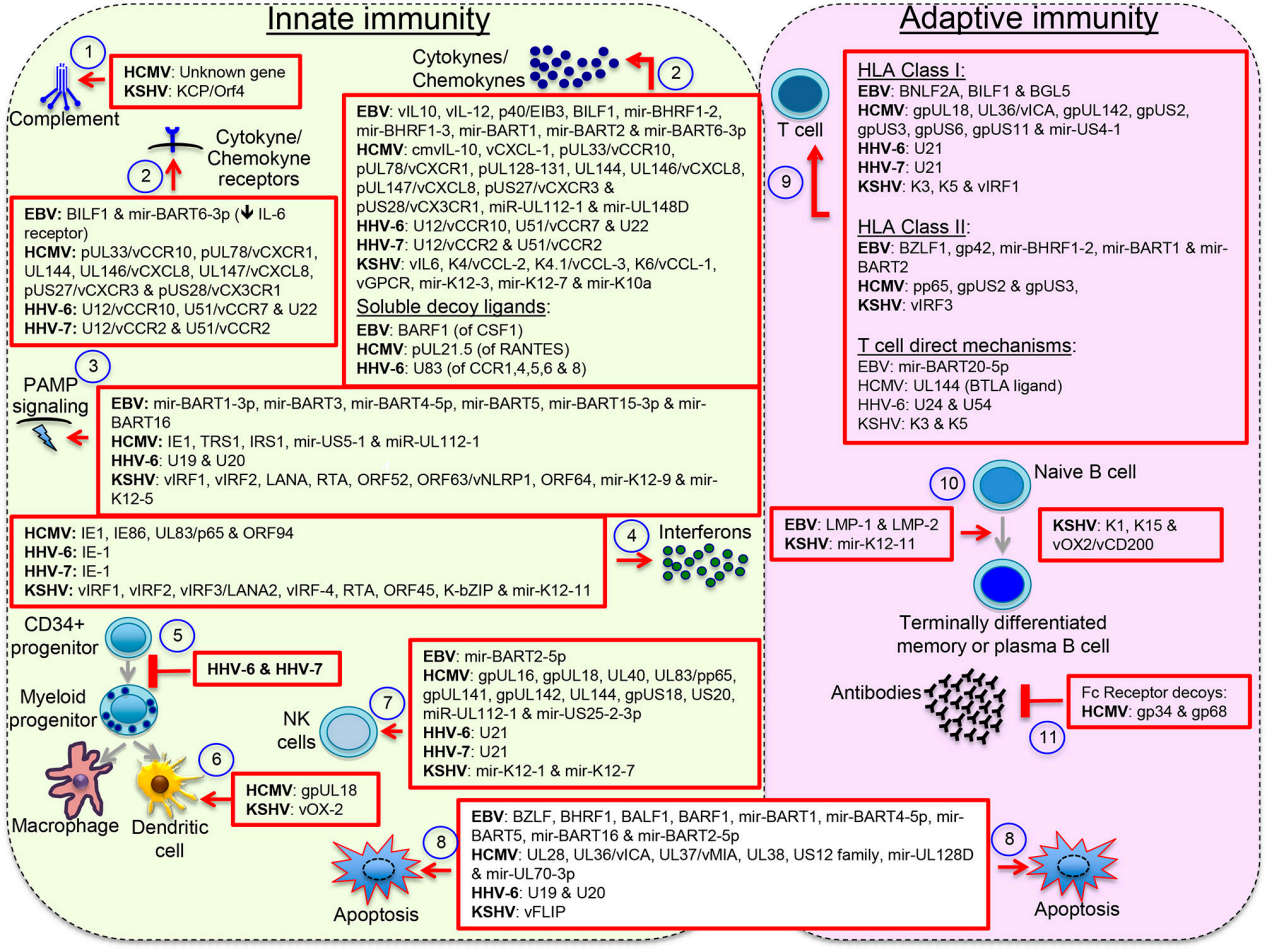
Herpesvirus immunomodulatory mechanisms. Immunomodulatory viral genes function at multiple levels: (1) antagonizing complement, (2) interfering with host cytokines and chemokines, (3) & (4) interfering with the pathogen-associated molecular pattern (PAMP) signaling pathways that lead to interferon activation or directly with the effects of interferons, (5) inhibiting maturation of myeloid lineages, (6) interfering with the activity of terminally differentiated myeloid cells, (7) interfering with expression or function of NK cell activating and inhibitory receptors, (8) blocking intrinsic and extrinsic mechanisms of cell apoptosis, (9) interfering with activation of T cells through inhibition of peptide processing or HLA/peptide molecules surface deposition or through direct mechanisms of T cell inactivation, (10) inducing B cell differentiation into long-lasting immunoprivileged memory B cells, and (11) interfering with humoral responses though expression of Fc receptor-like molecules. Other reported mechanisms not illustrated here include formation of regulatory populations and interference with the PD1/PDL1 and other immune regulatory checkpoint proteins. Viral orthologs of human cytokines/chemokine/receptors are denoted with the letter *v*. This figure does not pretend to be a complete review but to illustrate the wide spectrum of immunomodulatory mechanisms displayed by herpesviruses.

Herpesviruses have evolved with an arsenal of immunomodulatory proteins and miRNAs, with up to 30% of the herpesvirus genome made of genes with sequence similarity to cellular genes, of which half encode proteins that affect immune-related processes or control apoptosis of immune cell (Holzerlandt et al., 2002). Although the traditional interest on these gene orthologs relates to their capacity to counteract host responses in a pathological setting, both agonistic and antagonistic functions have been described. For instance, EBV expresses two viral proteins with the capacity to mimic B cell antigen receptor (BCR) signals (the latent membrane protein 2 or LMP-2A) and the CD40 signal (LMP-1), and according to the germinal center model, by mimicking these two immune cell receptors EBV is capable to expand infected naïve B cells in germinal center-like reactions and direct their differentiation into long-lasting memory B cells (Thorley-Lawson, 2015). LMP-1 and LMP-2A function illustrates the capacity of EBV to influence B cell maturation and perhaps influence global humoral responses too. Similarly, KSHV encodes miR-K12-11 a viral ortholog of cellular miR-155, which drives B cell expansion and differentiation, and has a critical role for the germinal center reaction and B cell terminal differentiation (Dahlke et al., 2012). KSHV also encodes proteins with ITAM (K1) and TRAF (K15) domains similar to LMP-2A and LMP-1. However, the relevance of these proteins for B cell differentiation and KSHV long-term persistence is not clear. Infection of CD34+ progenitor cells by beta herpesviruses inhibits their maturation into differentiated myeloid cell lineages, perhaps to promote persistence in progenitor cells, which together with memory cells are immunoprivileged sanctuaries. Some viral proteins concurrently exhibit opposite functions, for instance vIRF3/LANA2 activates IRF-3 & IRF-7 but inactivates IRF-5. Others exhibit multiple immunomodulatory functions, e.g., EBV BILF1 inhibits HLA class I presentation and CXCL12/CXCR4 signaling. T cell activity can be affected by inhibiting HLA class I and class II antigen presentation, but also more directly by upregulating checkpoint proteins or increasing sensitivity to apoptotic pathways; e.g., EBV miR-BART20-5p targets Tbet increasing T cell apoptosis and HCMV UL144 acts as a decoy ligand for checkpoint protein BTLA. See Figure 2 for an illustration of the extensive immunomodulatory mechanisms displayed by herpesviruses.

## Herpesvirus mutualistic relationship

Little is known about the course of the herpesvirus infection during clinically asymptomatic stages. Recent studies have shed light on the capacity of herpesvirus to influence immune responses to other pathogens, with evidence of advantages for hosts with a previous history of infection. For instance, γMHV68 and murine (M)CMV are murine models of EBV/KSHV and HCMV, respectively. Infection with these herpesviruses conferred protection against secondary infection by pathogenic bacteria (Barton et al., 2007). In the study by Barton ES et al., virally induced interferon (IFN)γ triggered systemic activation of macrophages preventing subsequent infection by pathogenic *Listeria monocytogenes* and *Yersinia pestis* (Barton et al., 2007). A mutant γMHV68 unfit to establish latent infection but lytic cycle competent was unable to activate macrophages and to confer protection. Similarly, in a previous study Selin LK et al. found that MCMV protected against vaccinia, lymphocytic choriomeningitis virus and Pichinde virus (Selin et al., 1998), and later studies also showed γMHV68 protection against type 1 adenovirus (Nguyen et al., 2008) and plasmodium (Haque et al., 2004).

The above-mentioned data support that murine herpesviruses promote immune cross-protection through increased levels of IFNγ. T cells are an important source of IFNγ and EBV and HCMV promote high frequencies of T cells (White et al., 2012). For instance, during IM about 50% of all peripheral CD8 T cells are directed against EBV (Callan et al., 1996; Scherrenburg et al., 2008), of which up to 10% remain present during persistent non-symptomatic infection (Hislop et al., 2002, 2005). Similarly, about 20% of blood memory CD4 and CD8 T cells are directed against HCMV in healthy carriers (Sylwester et al., 2005). It is possible that enhanced numbers of T cells (and perhaps of other immune cells such as NK cells and macrophages) are responsible for the cross-protection against IFNγ-sensitive pathogens. Also, herpesvirus infection can result in heterologous activation of T cells due to cross reactive antigens or to cytokines that indiscriminately influence T cell activation, expansion or differentiation, such as IL-15, IL-7, and IL-2 (Geginat et al., 2001). Intriguingly, heterologous activation of the immune system does not represent an universal mechanism of infection-mediated cross-protection as it is not observed after Pichinde virus infection (Selin et al., 1998), and upon vaccination against acute viruses, such as yellow fever or vaccinia, even though vaccination results in significantly increased T cell numbers (Liang et al., 2009). Also, infection with HSV-1 and HSV-2 does not increase T cells to similar levels than EBV and HCMV and does not induce cross protection (Barton et al., 2007; Sheridan et al., 2007). Of note, γMHV68 latent infection is associated with persistent high levels of RANTES, IL-6, TNFα and IFNα/β, additionally to IFNγ (White et al., 2012). Recombinant mice lacking expression of HOIL-1 or IL-6, and a Caspase-1/Caspase-11 double knockout exhibit an increased susceptibility to various pathogens. The hyper-inflammatory state promoted by γMHV68 alleviated that immunodeficiency (MacDuff et al., 2015).

NK cells are also expanded upon herpesvirus infection. Mice infected with MCMV exhibit an up to 100-fold expansion in the spleen and a 1,000-fold expansion in the liver, followed by a contraction of these robust numbers and formation of a long-lived pool of NK cells (Sun et al., 2009). Although part of the innate arm of the immune system, these residual NK cells exhibit a memory-like behavior, displaying recall-like responses characterized by a rapid and potent cytotoxic activity with enhanced release of IFNγ when re-stimulated with MCMV (Sun et al., 2009). This process is termed NK cell arming, and a similar memory-like NK cell pool has been observed in HCMV seropositive healthy individuals (Lopez-Verges et al., 2011). Armed NK cells potently respond to HCMV infected fibroblasts (Rolle et al., 2014) and are characterized by high expression of activating heterodimeric receptors CD94/NKG2C, lack of expression of the inhibitory receptor NKG2A and expression of the differentiation marker CD57 (Lopez-Verges et al., 2011). In HCMV seropositive healthy individuals, up to 90% of the peripheral NK cell pool is formed by memory-like cells (Holmes and Bryceson, 2016). Importantly, NK cell potent responses against hepatitis B virus (HBV), hepatitis C virus (HCV), human immunodeficiency virus (HIV), hantavirus, chikungunya virus and HSV-1 have been observed exclusively in HCMV seropositive individuals (Guma et al., 2006; Bjorkstrom et al., 2011; Petitdemange et al., 2011; Beziat et al., 2012; Zhang et al., 2013).

NK cells express a diverse repertoire of receptors of constitutive and facultative regulated expression. Activation of these cells is driven by a combination of activating and inhibitory signals in which the former outweigh the latter. The balance between activating and inhibitory receptor expression is importantly regulated by epigenetic modification, and two recent studies support that HCMV infection results in NK cells widespread epigenetic changes (Lee et al., 2015; Schlums et al., 2015). In the study by Lee et al HCMV infection promoted expansion of a heterogeneous NK cell population with various degrees of expression of signaling molecules, of which the FcγR/Syk negative population was highly responsive to CD16 crosslinking (Lee et al., 2015). Importantly, this NK population strongly responded to heterologous pathogens, e.g., influenza virus. Likewise, Schlums et al observed that in addition to FcγR/Syk, NK cells exhibited heterogeneous expression of EAT-2 and SAP (Schlums et al., 2015). In both studies promoter methylation correlated with levels of expression of Syk/EAT-2/SAP. Indeed, a hyper-methylated profile characterizes antigen-exposed CD8 T cells, which is similar to the profile found in memory-like CD57^bright^ NK cells. In conclusion, both studies support an HCMV-dependent switch of NK cell receptors and intracellular signaling proteins mediated by epigenetic regulation, in which FcγR & Syk are lost and CD16 is gained. Since the overall result of this switch is loss of FcγR (one ITAM domain) and gain of CD16 (three ITAMs because CD16 associates with CD3ζ), these data argue that reduced NK cell activating thresholds are favored upon HCMV exposure, which is also in agreement with previous observations about the memory-like NK cells replacing inhibitory (NKG2A) for activating (CD94/NKG2C) receptors (Lopez-Verges et al., 2011). The memory-like NK cell pool is long-time maintained, perhaps because of sporadic episodes of HCMV reactivation or by IL-15-dependent homeostatic expansion (Huntington et al., 2007; Schlums et al., 2017).

Chronic infection by γMHV68 not only cross-protects against heterologous pathogens but also against challenge with lymphoma cells (White et al., 2010), capacity that also correlated with enhanced NK cells cytotoxicity and increased IFNγ secretion. Also, γMHV68 infection protected against lupus-like autoimmune disease in a lupus prone murine strain (Larson et al., 2012), and against diabetes type 1 in non-obese diabetic (NOD) mice (Smith et al., 2007). Furthermore, infection also regulates homeostatic turnover and repair of epithelial tissue. Sun et al observed that chronic infection by MCMV and γMHV68 enhanced proliferation of epithelial cells, and this activity was attributed to a type I-IFN mediated enhanced expression of *apolipoprotein L9a* and *apolipoprotein L9b* and activation of the ERK MAP (mitogen activated protein) kinase (Sun et al., 2015). Overall, these studies support that murine and human beta- and gamma-herpesviruses improve immune cell defenses against other pathogens, as well as enhance tumor surveillance mechanisms, protect against autoimmune disease and perhaps influence many other host's homeostatic processes (Figure 1).

## Unbalanced virus-host interactions: increased susceptibility to EBV infection

Host-virus mutualistic or parasitic interactions are probably dynamic and influenced by a variety of different stimuli. For instance, in chronically infected plants this is shaped by environmental conditions (Roossinck, 2015b). Similarly, different circumstances can shift the herpesvirus/host equilibrium toward virus-induced malaise, of which delayed infection, immunosuppression and several primary immunodeficiencies (PIDs) are the best-characterized examples. EBV infection in children is mostly asymptomatic. However, 10% of infected adolescents or young adults develop IM. While IM usually have a self-limiting clinical course, some individuals develop a pathological EBV infection with a variety of life-threatening complications. In many cases this predisposition to EBV infection has been mapped to single-gene inborn errors (Palendira and Rickinson, 2015).

The classical definition of PID is the condition in which a single gene mutation generates a state of predisposition to multiple infectious agents, as observed in severe combined immunodeficiencies (SCID). However, in the last decades, other non-classical pathologies including exacerbated inflammation, autoimmunity, angioderma, hemophagocytosis, microangiopathies, and cancer are progressively been shown to result from inborn errors of immunity. Similar to classical PIDs, all these novel syndromes are inherited as Mendelian traits, but contrary to them, susceptibility to infection is usually limited to one microbial genus or even to a single species. These non-classical PIDs have extended our notion of immunodeficiency, paving the way for a better understanding of the genetic lesions and molecular mechanisms underlying the full spectrum of interactions between infectious agents and the immune system.

**Figure 3:**
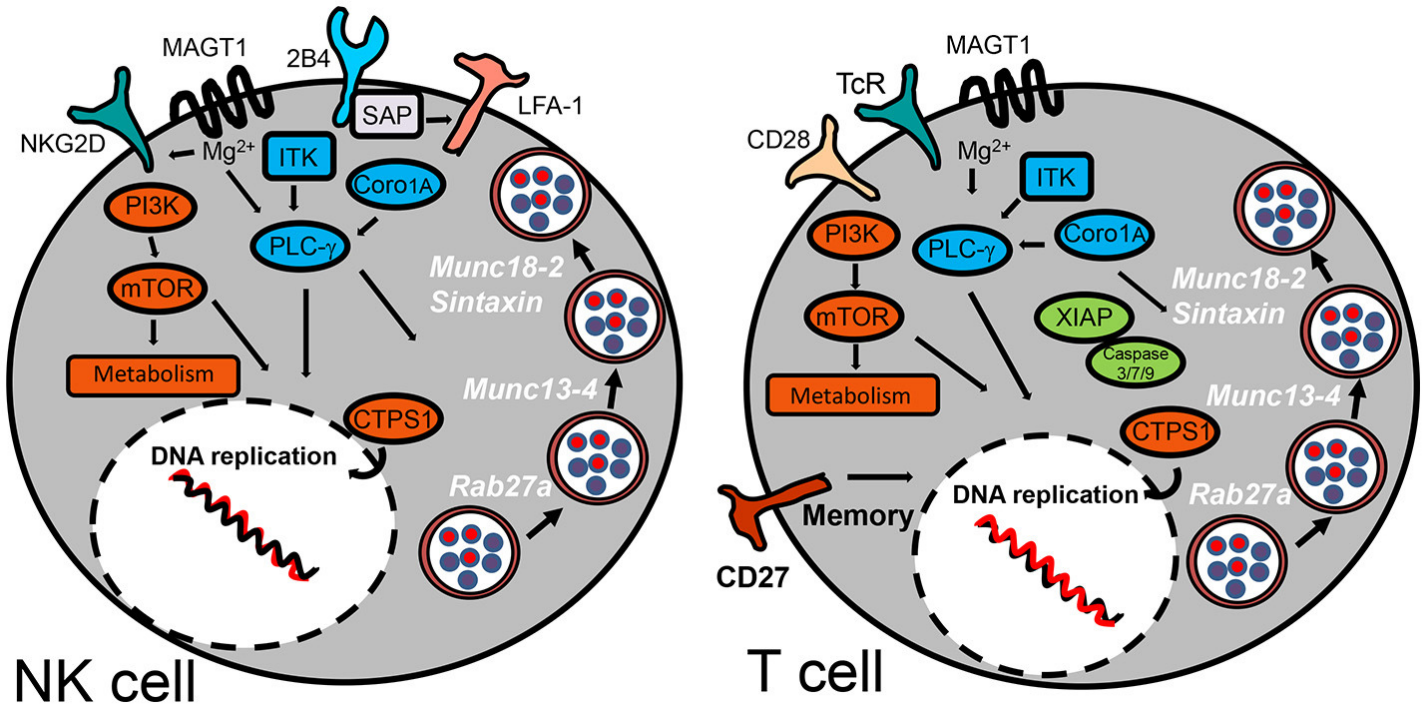
Inborn errors predisposing to EBV-morbidity by compromising NK and T cell cytotoxic activity. The schematic diagram highlights the cell surface receptors and signaling-pathway elements that are responsible to control infection for herpesviruses. Signaling proteins shown in blue are those involved in regulation of PLC-γ. Those shown in orange are implicated in regulation of metabolism. XIAP is the only signaling protein described hitherto implicated in regulation of apoptosis. SAP has been shown to influence cell-mediated cytotoxicity by promoting NK cell adhesion by an LFA-1-dependent mechanism. Cytosolic proteins regulating vesicular traffic of cytotoxic granules are indicated in white.

**Figure 4:**
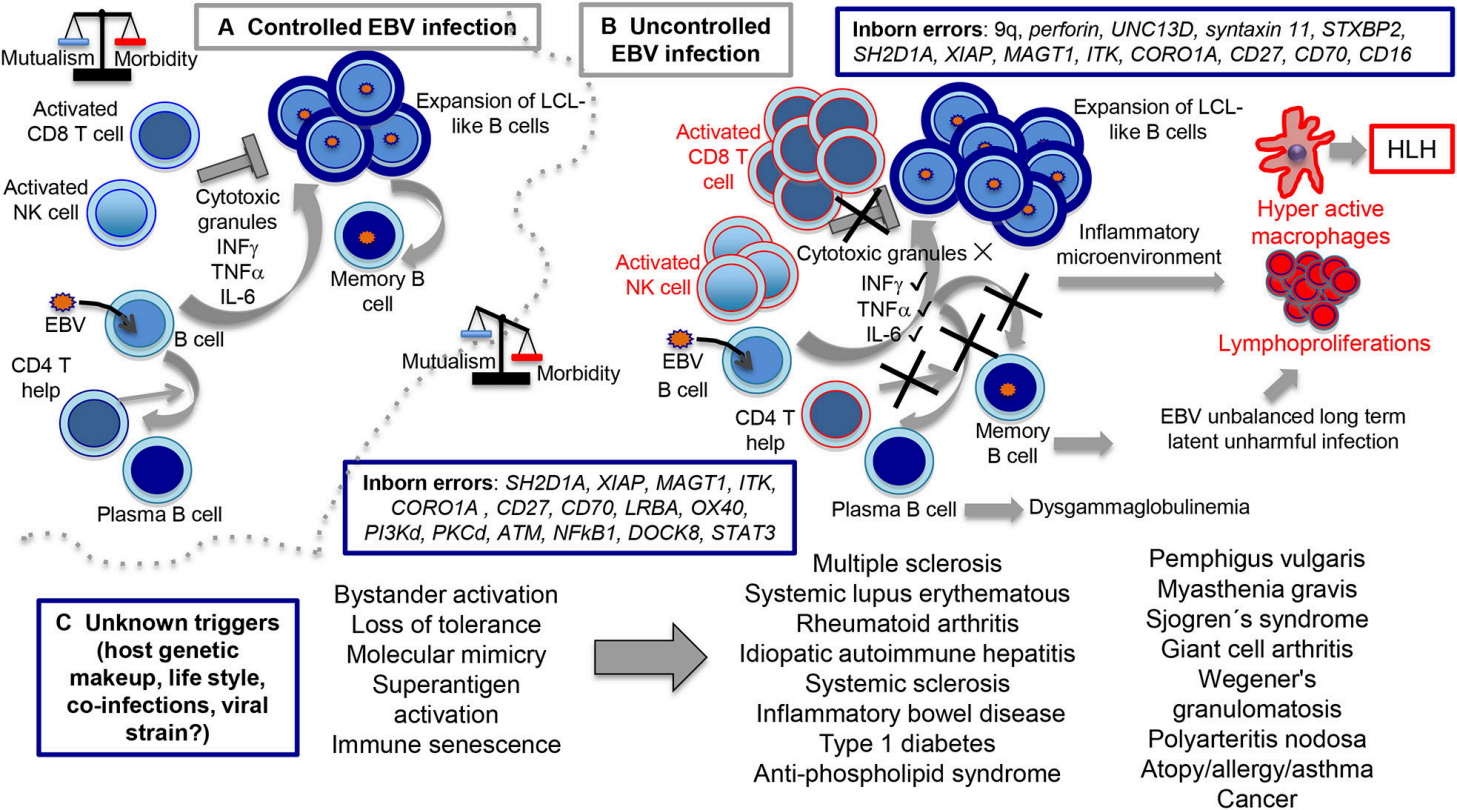
Routes of controlled and uncontrolled infection using EBV as example. In the **(A)** scenario, EBV has the capacity to induce B cell expansions through formation of germinal center-like reactions and lymphoblastoid-like cells (LCLs). At the end, *ad hoc* cytotoxic CD8 T and NK cell activities directed against the LCL-like cells forces EBV to downregulate viral expression, restricting infection to the memory B cell compartment in which a persistent infection is established. In the **(B)** scenario, inborn errors interfere with the cytotoxic activity (upper part), resulting in hyperactive CD8 T and NK cells still capable to secrete cytokines, such as IFNγ, TNFα, IL-6, and presumably many others, creating the inflammatory microenvironment responsible for macrophages hyperactivity, HLH and lymphoproliferation. On a collateral scenario (lower part of **B**), inborn errors that affect CD4 T cell function result in impaired T cell-B cell cooperation with diminished formation of terminally differentiated B cells and dysglobulinemia syndromes. Since EBV resides in memory B cells, we hypothesized that this condition unbalances the capacity of EBV to establish unharmful latent infections. In the **(C)** scenario, still unknown causes tip the balance toward disproportionate bystander activation, loss of tolerance, molecular mimicry, superantigen activation, immune senescence, and perhaps other undocumented mechanisms resulting in inflammatory/autoimmune diseases.

Up to 15 different genes have been described that, when mutated, usually lead to a persistent state of EBV viremia and severe immune dysregulation (Table 1). These PIDs with severe EBV disease manifest after primoinfection even in very young children. Disease results from either/both, the virus direct pathogenic mechanisms and the hyper-active immune response unable to control the infection. Two non-mutually exclusive groups of PIDs are observed based on these characteristics, familial forms of hemophagocytic lymphohistiocytosis (HLH) (a mainly immunopathological trait) and/or malignancy-associated lymphoproliferative disorders (an EBV directly induced trait). One of the hallmarks of these PIDs is the presence of chronically stimulated CD8 T cells and NK cells with impaired cytotoxic function (see below and Figures 3, 4). IM is also considered an immunopathological disease characterized by elevated peripheral numbers of NK and CD8 T cells directed against EBV antigens (Callan et al., 1996). Inborn errors compromising CD4 T cells can also confer increased EBV susceptibility. In total, there are about 40 genetic conditions in which life-threatening EBV-associated malaise has been reported; those occurring in a small proportion of patients are listed in Table 2.

**Table 1:** Inborn errors and genetic syndromes characterized by an increased susceptibility to EBV -associated morbidity.

**Gene mutated**	**Process affected**	**Type of inheritance**	**Myeloid lineage defects**	**NK/NKT cell defects**	**T cell defect**	**B cell defect**	**Virus involved**	**Frequency**	**Clinical symptoms**	**OMIM number**
*Perforin*	Cytotoxic cell-mediated lysis	Autosomal recessive	Hyper-active macrophages	Defective	Defective CTL	None	EBV HCMV	Common	HLH	603553
*UNC13D*	Cytotoxic granule release	Autosomal recessive	Hyper-active macrophages	Defective	Defective CTL	None	EBV HCMV	Common	HLH	608898
*STX11*	Cytotoxic granule release	Autosomal recessive	Hyper-active macrophages	Defective	Defective CTL	None	EBV HCMV	Common	HLH	603552
*STXBP2*	Cytotoxic granule release	Autosomal recessive	Hyper-active macrophages	Defective	Defective CTL	None	EBV HCMV	Common	HLH	601717
*SH2D1A*	NK and T cell adhesion and signaling	X-linked	None	Defective activation, NKT absents	Defective CTL	Reduced Memory	EBV	Common	HLH Lymphoma	308240
*BIRC4*	Inhibitor of apoptosis, Activator of NFκB	X-linked	None	NKT may be absents.	None	Reduced Memory	EBV	Often	HLH	300635
*MAGT1*	Mg2+ transporter, TCR signaling, NKG2D expression	X-linked	None	Defective NK cell activation	Defective CTL	Reduced Memory	EBV	Common	B cell Lymphoma	300853
*ITK*	TCR signaling	Autosomal recessive	None	Decreased NKT	Chronic T Lymphopenia	None	EBV	Often	B cell LfP	613011
*CD27*	TNFR-like signaling protein expressed on T cells and memory B cells	Autosomal recessive	None	Decreased NKT	None	Reduced Memory	EBV	Common	HLH	615122
*CD70*	CD27 ligand	Autosomal recessive	None	Decreased NKT	Reduced CD8+ T cells	Reduced Memory	EBV HCMV	Common	B cell LfP & lymphoma	Unlisted
*CORONIN1A*	Actin binding protein T cell egression of thymus	Autosomal recessive	None	Decreased NKT	Reduced CD8+ and CD4+ T cells	Reduced Low Ig	EBV	Common	B cell LfP	615401
*CTPS1*	Biosynthesis of phospholipids and nucleic acids	Autosomal recessive	None	Absence of iNKT	Reduced CD4+ T cells	Reduced Memory Dys Ig	EBV	Common	Recurrent reactivation Lymphoma	615897
*PI3Kd*	AKT-mTOR signaling pathway, metabolism	Autosomal dominant	None	Defective NKG2D-dependent activation	Reduced CD4+ T cells Senescent CD8+ T cells	Reduced Memory Dys Ig	EBV HCMV	Often	CAEBV B cell lymphoma	615513 616005
*CD16*	CD16 mediated-signaling	Autosomal recessive	None	Defective CD16-dependent activation	None	None	EBV	Common	CAEBV B cell LfP	615707
*MST1*	Serin/threonine kinase, pro-apoptotic protein	Autosomal recessive	Neutropenia	None	Reduced CD4+ T cells	Reduced	EBV HHV6	Often	B cell LfP & lymphoma	614868

*Common = ≥75% and Often = 50–75% of reported cases include some form of EBV dysregulation. Reference (Palendira and Rickinson, 2015) reviews in detail whether the susceptibility is exclusive to EBV or also to additional infectious agents. Data was taken from the Online Mendelian Inheritance in Man (OMIM) database (https://www.omim.org/) based on the original reports for each of the PIDs. Low Ig, hypogammaglobulinemia; Dys Ig, dysgammaglobulinemia (most often high IgM and low of other immunoglobulins), LfP, linfoproliferation*.

**Table 2:** Inborn errors and genetic syndromes in which there is dysregulation of EBV or of other herpesviruses.

**Gene mutated**	**Process affected**	**Type of inheritance**	**NK/NKT defects**	**T cell defect**	**B cell defect**	**Virus involved**	**Frequency**	**Clinical symptoms**	**OMIM numbers**	**PMID numbers**
LYST	Lysosomal trafficking	Autosomal recessive	Impaired NK cell activity	Impaired CD8 activity	None	EBV	Often	HLH, LfP	214500	2165746 3018035
Artemis	DNA repair VDJ recombination	Autosomal recessive	None	Polyclonal T cells	Reduced /absent	EBV	Often	B cell lymphoma	602450	12569164
ALPS^*^	Apoptosis and antigen signaling pathways	Autosomal dominant & recessive	None	Hyper-active	Reduced memory Increased B1	EBV	Seldom	CAEBV SLE-like AID NK and B cell LfP	601859 603909 607271 615559 614470 616100	23430113 25374737 21626105
MCM4	DNA repair	Autosomal recessive	Defective	None	None	EBV HCMV	Seldom	B cell LfP	609981	22354167
IL2RG	IL2-receptor	X-linked	Reduced	Reduced	Reduced	EBV	Seldom	B cell LfP	300400	22936741
GATA2	Hematopoietic TF	Autosomal dominant	Reduced	Reduced CD4+ T cells	Reduced	EBV HCMV	Seldom	Smooth muscle cell sarcoma HCMV reactivation	614172	23728141
IL-10/IL-10R	Anti-inflammatory Cytokine & receptor	Autosomal recessive	Normal to Hyper-active	Normal to Hyper-active	Normal	EBV HCMV	Seldom	HCMV reactivation B cell LfP	146933 612567	23158016
LigIV	DNA ligase, DNA repair	Autosomal recessive	Reduced	Multiple cytopenias	Multiple cytopenias	EBV	Seldom	B cell lymphoma	601837	16585603 17345618
ATM	DNA damage response	Autosomal recessive	None	Reduced	Reduced memory	EBV	Seldom	B cell lymphoma	208900	25488969 20380744
WAS	Cytoskeleton rearrangement protein downstream of CDC42	X-linked	Reduced activity	Reduced	Reduced	EBV KSHV HCMV	Seldom	HLH B cell lymphomas KS HCMV reactivation	301000	12892170 20429642 4584223
RAB27A	Chemoquine receptor	Autosomal recessive	Reduced activity	Reduced	None	EBV HHV6	Seldom	HLH	607624	22111599 19953648
CXCR4 (WHIM)	Leukocyte migration	Autosomal dominant	None to reduced	None to reduced	Reduced	EBV	Seldom	B cell lymphoma HLH T cell LfP	193679	12224006
TBX1	TF regulates thymus development and stem cell quiescence	Autosomal dominant	Reduced	None	None	EBV	Seldom	B and T cell lymphoma CAEBV AID	188400	21683977 10519724
NFκB1	TF regulates immune responses	Autosomal dominant	None	Impaired	Reduced memory	EBV	Seldom	B cell LfP Low Ig AID	616576	27338827
DOCK8	Interacts with Cdc42 and regulates actin	Autosomal recessive	None	Reduced CD4	Reduced memory	EBV CMV	Seldom	LG B cell LfP	243700	27890707 28293550
STAT3	TF activated by cytokine signaling	Autosomal dominant	None	Reduced CD4	Reduced memory	EBV	Seldom	B cell lymphoma	147060	22118528 20859667
LRBA	LPS-mediated vesicle trafficking in B/T cells	Autosomal recessive	Low	Reduced CD4	Reduced	EBV	Seldom	B cell LfP Low Ig	614700	22721650
ZAP70	Kinase participates in TCR signaling	Autosomal recessive	None	No CD8 Impaired CD4	Reduced	EBV	Seldom	B cell lymphomas Normal to increased IgE	269840	21094993
CARD11	Participates in NFκB signaling	Autosomal recessive	None	Impaired Reduced Tregs	Reduced	EBV	Seldom	CAEBV AID Low Ig	615206	23129749
SP110 (VODI)	Leukocyte specific nuclear body protein	Autosomal recessive	None	Reduced	Reduced	HCMV	Seldom	HCMV reactivation HCMV-colitis Low Ig	235550	22621957
MALT1	Activates NFκB signaling	Autosomal recessive	None	Impaired	Impaired	HCMV	Seldom	HCMV reactivation HCMV pneumonitis	615468	25627829
NEMO	Activates NFκB signaling	X-linked	None	Impaired	Reduced	HCMV	Seldom	HCMV sepsis	300248	15100680
STIM1/ORAI1	Antigen receptor signaling	Autosomal recessive	None	Impaired	Impaired	HCMV KSHV	Often	KS	612783	22190180 20876309
INFRG1	Receptor of IFNγ	Autosomal recessive	None	Reduced activity	None	HCMV KSHV	Seldom	KS HCMV sepsis	209950	15069403 10547254
OX40/CD34	TNFR superfamily Co-stimulatory checkpoint protein of T cell activation	Autosomal recessive	None	Reduced	Reduced memory	KSV	Often	KS	615593	20156905 23897980

Often = 50–75%, Seldom = ≤ 50% of reported cases. Data was taken from the Online Mendelian Inheritance in Man (OMIM) database (https://www.omim.org/) based on the original reports for each of the PIDs. PMID number is the accession number(s) of examples of studies in which a link between morbidity and herpesvirus infection has been reported in the pubmed database (https://www.ncbi.nlm.nih.gov/pubmed). LYST (Chediak-Higashi syndrome), ALPS (Autoimmune Lymphoproliferative Syndrome), GATA2 (MonoMAC syndrome), LigIV (LigIV Syndrome), ATM (Ataxia telangiectasia), WAS (Wiskott Aldrich syndrome), RAB27A (Griscelly syndrome type 2), WHIM (Warts hypogammaglobulinemia immunodeficiency and myelokathexisis syndrome), TBX1 (DiGeorge syndrome), DOCK8 or STAT3 (Hyper IgE syndrome), VODI (Hepatic veno-occlusive disease). AID, autoimmune disease, LfP, linfoproliferation; KS, Kaposi sarcoma; TF, Transcription factor; LG, Lymphomatoid granulomatosis; Low Ig = hypogammaglobulinemia, ALPS^*^ = multiple genes involved [TNFRSF6 (FAS); TNFSF6 (FAS ligand), CASP10, CASP8, PRKCD, RALD, CTLA4].

### Immunodeficiencies impairing NK and CD8 T cell cytotoxic function

HLH is a life-threatening complication characterized by an exaggerated activation and expansion of T/NK lymphocytes and hemophagocytic macrophages. Those cases in which an inherited genetic cause is known have been categorized as primary or familial (FHL). Five genetic defects have been identified for FHL, of which one on chromosome 9q (FHL-1 or FHL type 1) still does not have a specific gene linked to the disease. Mutated genes associated with FHL-2 (*Perforin*), FHL-3 (*UNC13D*), FHL-4 (*Syntaxin11*), and FHL-5 (*STXBP2*) encode for proteins that are related to NK and T cells cytotoxic activities. Cytotoxic CD8 T cells (CTLs) and NK cells contain cytoplasmic granules rich in lytic enzymes such as perforin and granzymes, which are essential for their cytotoxic activity. Upon engagement of activating receptors, cytotoxic granules undergo regulated secretion of their content into the synaptic secretory cleft. This process is highly coordinated and characterized by a series of discrete steps involving granule biogenesis, polarization, maturation, fusion with plasma membrane, and recycling (Bryceson et al., 2012). There are other genetic causes impairing granule secretion, such as Chediak-Higashi and Griscelli type 2 syndromes (Sepulveda and de Saint Basile, 2017) (see Table 2). These syndromes affect many types of immune cell, besides NK and CTLs, resulting in high susceptibility to a wider spectrum of infectious agents; although, patients sometimes also develop EBV viremia and HLH.

Although, up to 74% of infection-triggered HLHs can be linked to EBV primoinfection or reactivation (Trottestam et al., 2011; George, 2014), HCMV, and more rarely HHV-6 and KSHV, have also been linked to HLH. FHLs provided direct evidence of the importance of the cytotoxic function of T/NK cells to control immune homeostasis during EBV infection (Henter et al., 1993). However, the precise mechanism by which a deficient cytotoxic activity underlies HLH remains unknown. The most accepted explanation is based on the inability of cytotoxic cells to remove infected cells, the source of antigenic stimulation. Hence, T cells are persistently activated secreting high levels of pro-inflammatory cytokines IFNγ and TNFα. This condition leads to a sustained tissue infiltration of activated macrophages that also results in persistent high levels of IL-6, IL-18, and more TNFα, cytokines that are responsible for various of the clinical and immunological manifestations observed in patients with HLH. Alternatively, another non-mutually exclusive mechanism can be envisioned. Activated immune cells are eliminated to some extend by cytotoxic cells during the contraction phase after infection resolution. As a consequence of impaired cytotoxic activity, activated T and NK cells may not properly eliminate themselves.

X-linked lymphoproliferative (XLP) syndromes are PIDs characterized by an extreme vulnerability toward EBV infection that includes a fulminant form of IM, dysgammaglobulinemia and lymphoma, frequently co-occurring with HLH (Filipovich et al., 2010). The first (XLP-1) was defined by mutations in *SH2D1A* that encodes the SLAM adapter SAP (Coffey et al., 1998; Nichols et al., 1998; Sayos et al., 1998), while XLP-2 is caused by mutations in the gene encoding the X-linked inhibitor of apoptosis protein (XIAP) (Rigaud et al., 2006). SAP is expressed in T, NK, NKT, and some B cells. Recent studies suggest that the SAP adapter proteins are molecular switches that control SLAM receptors interactions with inhibitory or activating signaling pathways (Veillette et al., 2009). Initial studies from XLP-1 patients suggested that SLAM receptors (such as CD244/2B4) were critical cytotoxic cell receptors responsible for killing EBV-infected B cells. In agreement with this hypothesis, absence of SAP leaves 2B4 unfit to generate a positive signal for cell-mediated cytotoxicity. Unlike XLP-1, XIAP deficient patients do not develop lymphomas and instead present an increased risk for chronic hemorrhagic colitis. XIAP is ubiquitously expressed in different tissues including immune cells, in which it regulates apoptosis by inhibiting caspases 3, 7, and 9. XIAP is also involved in regulation of NFκB and MAP kinase signaling (Krieg et al., 2009). The mechanisms underlying the pathophysiology of XLP-2 and link to EBV disease are poorly understood.

Mutations in genes encoding for signaling proteins that directly or indirectly regulate PLCγ activation also affect NK and T cell function or numbers, e.g., the magnesium transporter MAGT1, the IL-2-inducible T cell kinase ITK and Coronin 1A. TCR (T cell receptor) engagement triggers a transient increase in Mg2+ flux, and loss of this flux in MAGT1 deficiency negatively impacts on TCR-induced PLCγ activation. MAGT1 is also required to maintain NKG2D expression, an activating receptor of NK cells and CTLs (Chaigne-Delalande et al., 2013). Accordingly, a reduction in NKG2D expression correlates with their impaired cytotoxic function. Since MAGT1 and SAP deficient patients cannot control EBV infection, it is likely that both 2B4 and NKG2D receptors can synergize for proper cytotoxic anti-EBV responses, also preventing lymphoma development. ITK is also a key component of the TCR signaling complex that leads to PLCγ activation (Horwood et al., 2012). Likewise, T cells from patients carrying mutations in *ITK* show absent or low proliferation in response to CD3 stimulation. As seen for other patients suffering from susceptibility to EBV, NKT cells are severely reduced in ITK deficient patients. Coronin-1A associates with actin, connecting the plasma membrane to the cytoskeleton, activating vesicular trafficking, signal transduction, cell migration and/or phagocytosis in response to extracellular signals (Moshous and de Villartay, 2014; Punwani et al., 2015a). Coronin-1A is involved in T cell egression from thymus, regulation of CD4 T cell development and T cell death. However, recent studies support that it is also involved in PLCγ1 activation, and defects in lymphocyte survival and migration are better explained by the unpaired PLCγ1 signaling than by a cytoskeleton-dependent mechanism (Mueller et al., 2008). Interestingly, some patients also have a reduced number of NK cells with reduced cytotoxic function. Studies with *CORO1A* knocked down NK cell lines showed that depolymerization of F-actin caused cytotoxic vesicles to be inappropriately positioned at the immune synapse resulting in impaired degranulation (Mace and Orange, 2014).

CD27 and CD70 are transmembrane proteins, members of the TNF receptor superfamily that serve as ligand/receptor partners, and their reciprocal engagement regulates differentiation, survival and function of T, NK, and plasma/memory B cells (van Montfrans et al., 2012; Salzer et al., 2013; Tangye et al., 2017). CD27 PIDs almost always lead to EBV viremia and lymphoproliferation with an increased risk to develop B cell lymphoma, with some patients also developing HLH. The clinical and immunological features of CD70 deficiency are in general a phenocopy of those triggered by CD27 deficiency. Interestingly, while CD27 deficiency shows a high mortality rate (29% of patients die between 2 and 25 year of age), CD70-deficient individuals are clinically stable, suggesting that CD27 has CD70-independent functions. It has been hypothesized that CD27 is important for the generation of EBV specific memory cytotoxic effector cells; hence, lost of CD27 may affect the ability to control EBV reactivation (Alkhairy et al., 2015). Whether CD27 deficiency also affects NK cell effector functions is not presently known. CD16 is also a transmembrane protein and is the low affinity IgG Fc receptor. CD16 is an activating receptor and one of the most common markers used to identify NK cells. NK cell numbers are normal to low in CD16 deficient patients, with a significant decrease in their natural cytotoxicity. Patients with CD16 deficiencies often develop IM and EBV chronic viremia that leads to B cell lymphoproliferations (de Vries et al., 1996; Jawahar et al., 1996; Grier et al., 2012).

CTP synthase 1 (CTPS1) and phosphoinositide 3-kinase delta (PI3Kδ are two proteins that give us a clue on how cell metabolism is critical for immune control. CTPS1 is a cytidine nucleotide synthase involved in DNA and RNA synthesis, which is expressed at low levels in resting lymphocytes and strongly upregulated upon antigen-receptor engagement. *In vitro* studies confirmed the impaired ability of CTPS1 deficient T cells to proliferate upon activation (Martin et al., 2014). All patients carrying mutations in *CTPS1* exhibited an early onset of severe and persistent viral infections, mostly caused by EBV, VZV and encapsulated bacteria. In the initial report, two out of eight patients developed an EBV B cell non-Hodgkin lymphoma (Martin et al., 2014). A defect in NK cell proliferation was less dramatic but one patient exhibited complete absence of inducible NKT cells (Martin et al., 2014). More than 100 patients with activated PI3Kδ syndrome (APDS) have been described. APDS results from heterozygous gain-of-function mutations in *PIK3CD* or *PIK3R1*. PI3K is a family of heterodimeric signaling proteins composed of a p85 regulatory subunit and a P110α/β/δ catalytic subunit. All Mutations in P110δ result in an increased kinase activity, either by disrupting inhibitory contacts between regulatory p85 and p110δ or by increasing the affinity of p110δ for the plasma membrane. As consequence, T cells from patients with APDS exhibit augmented AKT phosphorylation and increased activity of mTOR, master regulators of cell survival and protein synthesis pathways. Gain-of-function mutant PI3Ks are hyper-activated upon T cell activation, resulting in increased sensitivity to TCR-induced T cell death and increased T cell senescence. One of APDS major clinical manifestations is severe and persistent herpesvirus infection with a high risk to develop EBV positive lymphadenopathies and B cell lymphomas.

### Immunodeficiencies impairing CD4 T cell-B cell cooperation

A progressive decrease in CD4 T cells and dysglobulinemia are observed in some ITK deficient patients concomitant with an increased CD8 T cell count (Horwood et al., 2012). Likewise, CD27, CTPS1, and ALPS deficient patients sometimes present low numbers of memory B cells and defective humoral responses, suggesting a defect in T cell-dependent B-cell function (Agematsu, 2000; Martin et al., 2014). For instance, ALPS patients exhibit elevated IgM, variable levels of IgG and reduced IgA. Because CD27 is one of the most widely used biomarkers to identify memory B cells (Agematsu, 2000), it is presently unclear whether the impaired humoral response results from lack of T cell help to activate B cells and/or an intrinsic defective B cell terminal differentiation. CD4 T cell deficiency and dysglobulinemia are both indicative of an altered B cell terminal differentiation. Since EBV resides in memory B cells in a quasi-dormant state in healthy individuals, a diminished capacity to form memory B cells may unbalance the virus-host mutualistic equilibrium affecting the ability of EBV to establish unharmful infections (see Figure 4). Of note, this scenario supports that EBV induced B cell maturation into memory B cells relays in more than viral products, e.g. LMP-1 and LMP-2A expression, also requiring some form of CD4 T cell help.

### PIDs associated with morbidity by other herpesviruses

KSHV is well known as the causative agent of KS, an angiogenic-inflammatory neoplasm that originates from endothelial cells, and of two B cell lymphomas MCD and PEL. These pathologies probably point out to the main cell lineages that act as reservoirs of KSHV latent and persistent infection. KSHV associated neoplasms are prevalent in individuals with acquired immunodeficiency because of HIV infection or immunosuppression after organ transplantation (see below). Idiopathic cases of KS are mostly reported in the Mediterranean regions of Africa, Asia, and Europe. KS is typically observed in the elderly in immunocompetent individuals, and extremely rare in childhood, with no more than 30 cases reported since 1960. Therefore, it has been hypothesized that host genetic factors may account for predisposition to symptomatic KSHV infection in children. Accordingly, four genes, *IFNGR1, WAS, STIM1*, and *OX40/TNFRSF4/CD34*, have been described as genetic etiologies underlying susceptibility to aggressive KSHV infection leading to KS (Jackson et al., 2016). It is proposed that these genetic defects impair T cell function (Byun et al., 2010), and that T cell function is required to control KSHV-infected endothelial cells (Byun et al., 2013). However, it is important to notice that contrary to inborn errors predisposing to EBV aggressive disease, development of lethal KS is not the primary manifestation associated with those mentioned PIDs, arguing for additional unknown co-factors. Also, all reported PIDs with increased susceptibility to KSHV do not show a concomitant increased frequency of MCD or PEL, similar to the profile observed in CD4 depleted HIV positive patients. Again, arguing for multifactorial causes of KSHV aggressive morbidity, in which genetic errors, but also co-infections, such as the one with HIV, may play critical roles. Supporting that scenario, in about 70% of PEL patients, lymphoma cells are co-infected with KSHV and EBV. However, since significantly less people are infected with KSHV, it is not clear whether a higher worldwide prevalence would result in more KSHV-morbidity.

Inborn errors predisposing to EBV and KSHV also frequently include an increased frequency of HCMV reactivation, HCMV sepsis and damage to the tissues harboring the virus (see Tables 1, 2). A few genetic errors predispose to aggressive HCMV infection independent of other herpesvirus co-morbidity; an example is *MALT1* mutations for which HCMV viremia and HCMV-related pneumonia have been reported (Punwani et al., 2015b). Another example is hepatic venoocclusive disease with immunodeficiency (VODI), a *SP110* deficiency characterized by hepatic vascular occlusion and fibrosis and susceptibility to multiple infectious agents, of which HCMV is one of the most commonly found associated with the hepatic lesion, but also with oropharyngeal and vulval disseminated infection (Cliffe et al., 2012). NFκB essential modulator (NEMO) deficiency also leads to HCMV sepsis (Orange et al., 2004). Like KSHV susceptibilities, patients with these inborn errors present a wide range of aggressive infections both viral and bacterial. To our knowledge, inborn errors predisposing to recurrent clinical manifestations with the roseolovirus have not been documented, perhaps because we still lack behind in the comprehension of these viruses molecular biology and associated diseases. However, the similarity to other herpesvirus, specially to HCMV, and the new pathologies added to these viruses beyond high fever and rash (such as encephalitis), most probably will uncover PIDs in which these viruses influence the spectrum of pathological symptoms.

### Chronic active EBV infection (CAEBV)

The term “chronic mononucleosis syndrome” was used to describe an atypically persistent IM. Today, CAEBV is defined as a life-threatening disease, characterized by exacerbated EBV infection (e.g., elevated antibody titers and high viremia). Beside IM-like symptoms, CAEBV patients often develop HLH and autoimmune-like manifestations in the form of skin rash and hypersensitivity to mosquito bites (Kimura et al., 2003). Unlike the classical EBV tropism for B cells, both NK cells and T cells are often infected in CAEBV patients, with risk of clonal expansion and development of NK or T cell lymphomas. Because of these increased numbers of EBV-infected lymphocytes, it has been suggested that CAEBV is more a lymhoproliferative disorder than an infectious disease (Kawa, 2000). However, it is not clear what triggers the lymphoproliferation and the altered tropism for NK and T cells. Based on similitudes with other well-described genetic errors leading to lymphoproliferative disorders, the search for a CAEBV genetic etiology has been conducted, mostly with negative results. However, in the study by Nomura et al., patient's with CAEBV were found to carry mutations in FAS, similar to the ones responsible for autoimmune lymphoproliferative syndromes (ALPS) (Nomura et al., 2011). Therefore, the possibility that single-gene errors are causative of CAEBV should not be disregarded.

### Immunosuppression

Life-threatening B cell lymphoproliferations are relatively common in immunosuppressed individuals because of HIV/AIDS or post-transplant therapy. These B cell expansions are related to the virus capacity to influence B cell proliferation, survival and differentiation, which efficiently result in B cell immortalization upon *in vitro* infection. Although polyclonal in the beginning, without medical intervention these B cell expansions can become true monoclonal lymphomas, which closely resemble lymphomas observed in immunocompetent hosts. Similar to PIDs, one of the main triggers of post-transplant lymphoproliferations is lack of the cancer immunosurveillance mechanisms mediated by CD8 T cells. In the case of HIV this is not as clear, since individuals in successful anti-HIV regimes with normal T cell counts are still at high risk to develop EBV positive lymphomas. This is unlike KSHV-mediated KS, MCP, or PEL that practically never occur in HIV controlled patients. We do not understand the mechanism by which HIV long-time carriers are at increased risk to develop EBV positive lymphomas, but HIV-EBV cellular or molecular interactions have been proposed. EBV infection and transformation of smooth muscle cells has also been observed in HIV-immunosuppressed patients. The reason of such altered EBV tropism is unknown, but GATA2 PIDs with severe NK cell deficiency sometimes also result in similar EBV positive sarcomas (see Table 2; Cohen et al., 2016).

## Autoimmune/inflammatory disorders linked to herpesvirus chronic infection

There are over 80 autoimmune diseases (AID), most of them of unknown etiology but that usually arise concomitant to inflammation. Viral infection and particularly EBV infection, is considered one of the most likely triggers. Among the AIDs with the stronger link with EBV are: multiple sclerosis (MS), systemic lupus erythematous (SLE), rheumatoid arthritis (RA) and idiopathic autoimmune hepatitis (IAIH); but there is also some evidence for systemic sclerosis (SSc), inflammatory bowel disease (IBD), type 1 diabetes, anti-phospholipid syndrome, pemphigus vulgaris, myasthenia gravis, Sjogren's syndrome, giant cell arthritis, Wegener's granulomatosis, polyarteritis nodosa, atopy and allergy (Barzilai et al., 2007; Temajo and Howard, 2014). There is also evidence for an HCMV association with SLE and SSc, and HHV-6 with MS and Hashimoto's thyroiditis (Halenius and Hengel, 2014). Most of this evidence comes from epidemiological studies in which increased antibody titers and/or increased seroprevalences are preferentially found in diseased individuals (White et al., 2012). Particularly important is the correlation of EBV serology with pediatric cases of SLE and MS, for which the seroprevalence of the population is lower (James et al., 1997). Higher anti-EBV antibodies (Farrell et al., 2009) and elevated levels of EBV BHRF miRNAs (Wang et al., 2017) also identify the most severe cases of MS. SLE patients also present with up to 40-fold higher EBV load in peripheral blood (Kang et al., 2004). However, it is important to consider that unambiguous causality is difficult to establish because of the complexity of these diseases, the ubiquity of infection in the population, and since the normal residence of the herpesvirus is the immune system, it is unclear whether the exacerbated infection is causative or a consequence of the disease. Furthermore, many studies included patients on immunosuppressive therapy, which could potentially allow for increased viral reactivation. Stronger causal association can be driven after clinical intervention, for instance, a patient with progressive MS was infused with CD8 T cells directed against EBV-latency antigens showing reduction of the clinical symptoms (Pender et al., 2014).

MS is a chronic inflammatory/neurodegenerative disease, in which immune cells trigger the appearance of central nervous system (CNS) demyelinating lesions. MS exhibits a geographical distribution that well correlates with that of IM, and IM increases more than twice the risk of MS (Thacker et al., 2006). In a series of longitudinal studies carried out in children and young adults, those that developed MS were found exclusively within the group that seroconverted and none in the group that remained EBV negative (Levin et al., 2010). On the contrary, there is not link between IM and SLE. SLE is characterized by systemic inflammatory manifestations with auto-antibodies directed against nuclear RNA-associated proteins Sm and Ro, and dsDNA. Tissue deposition of antigen-antibody complexes is one of the main causes of organ damage. A longitudinal study with the repository sera of the US Department of Defense also found the SLE cases almost exclusively in the EBV infected individuals, and EBV infection always preceded the appearance of auto-antibodies (McClain et al., 2005). Similarly, RA patients present high viremia. RA is characterized by the presence of auto-antibodies directed against citrullinated proteins. EBV infected plasma cells are present at synovial joints of RA patients (Blaschke et al., 2000), and often exhibit specificity toward citrullinated antigens (Croia et al., 2013). Joints of RA patients also present increased numbers of EBV specific CTLs, which may cooperate with local inflammation (Scotet et al., 1996; Tan et al., 2000). IAIH is a disease in which hepatic damage and elevated hepatic enzymes in blood co-exist with auto-antibodies. Also important is that the definition of IAIH includes lack of participation of known causes of hepatitis, e.g., infection with hepatotropic viruses or alcohol consumption. Besides the common presence of auto-antibodies, IM, CAEBV and HLH generally course with hepatomegaly and elevated hepatic enzymes, arguing that hepatic damage is common in EBV symptomatic infections. Furthermore, fatal cases of IM and CAEBV are often due to hepatic failure, and fatal cases of acute IAIH present with a high EBV load in liver (Papatheodoridis et al., 1995; Cacopardo et al., 2003; Palazzi et al., 2003; Chiba et al., 2004; Sanchez et al., 2008). In a longitudinal study by Vento et al. of relatives of IAIH patients, those that developed the disease were always preceded by IM (Vento et al., 1995), thus suggesting that EBV-related IAIH occurs in genetically susceptible individuals.

Strong evidence also supports a link between HHV-6 (mainly for HHV-6A) and MS. Besides the serological data, increasing viral loads are observed in MS patients, which closely correlate with episodes of disease relapse (Chapenko et al., 2003). MS brain lesions exhibit evidence of astrocytes and oligodendrocytes actively/lytically infected with HHV-6 (Goodman et al., 2003). Direct infection of thyrocytes, rather than of immune cells, has also been observed in patients with Hashimito's thyroiditis (Caselli et al., 2012). *In vitro* studies indicated that HHV-6 infected thyroid cells are targeted by NK cells, which may be a source of pathological damage and local inflammation (Caselli et al., 2012). Whether astrocytes, oligodendrocytes and thyrocytes are part of the normal tropism of HHV-6 is not clear, or similar to EBV infection of T and NK cells in CAEBV, and of smooth muscle cells in CD4 immunosuppression or GATA2 PID, unknown circumstances promote/sustain infection in atypical cells.

Different mechanisms have been proposed to explain the pathological roles of the herpesvirus in AIDs, beyond the potential for direct infection of CNS and thyroid cells. The great immunomodulatory capacity of these viruses may result in enhanced B, T, and NK cell numbers as it is observed in EBV and HCMV seropositive individuals, and in bystander activation of these cells and other immune cells. Latently infected cells could be attracted to the AID lesion because of an ongoing initial inflammation, and once there, viral expression together with local cytotoxic responses could aggravate the inflammatory process. Evidence exists of increased EBV infected B cells together with T cells directed against EBV antigens infiltrating MS lesions and synovial fluid of RA patients (Serafini et al., 2007). There is also evidence of rhesus CMV directing promiscuous T cell responses against immuno-dominant class II presented antigens (Hansen et al., 2013). Increased local cytotoxic activity also results in increased secretion of IFNs, TNFα and other immune molecules known to have a participation in several AIDs. EBV exosomes containing non-coding RNAs are also potent inducers of TLR activation in immune cells infiltrating AID lesions, and EBER expression correlates with MS areas of IFNα-enriched expression (Tzartos et al., 2012). Furthermore, viral orthologs of cytokines/chemokines and their receptors could indiscriminately activate innate and adaptive immune responses, altogether favoring the initiation or progression of inflammatory/autoimmune morbidity.

Most AIDs are characterized by the presence of self-reactive antibodies that are one of the main culprits of direct tissue damage in local AIDs, or of affecting vital organs because of immune complex deposition in systemic AIDs. EBV and KSHV can potentially break mechanisms of peripheral B cell tolerance through LMP-1/LMP-2A and miR-K12-11 expression. These viral products expressed in infected B cells could drive formation of germinal center-like cells, inducing terminal differentiation irrespective of the specificity of the BCR (Figure 2). Also, infected auto-reactive T and B cells may overcome negative selection mechanisms because of anti-apoptotic viral proteins/miRNAs. Altogether, this would result in formation of fully competent terminally differentiated self-reactive lymphocytes. Bacterial and viral super-antigens can also trigger polyclonal activation of auto-reactive adaptive cells. Particularly, EBV LMP-2A and HHV-6 have been shown to drive expression of a human endogenous retrovirus (HERV)-K18 super-antigen, which has been linked to several AIDs, including MS and type 1 diabetes (Hsiao et al., 2009; de la Hera et al., 2013).

Another extensively studied mechanism of AID immunopathology is shared antigenicity between viral and cellular proteins. This mechanism of molecular mimicry has been extensively reported for EBV EBNA-1, and anti-EBNA-1 antibody titers well correlate with MS presentation (Munger et al., 2011). Serum of SLE patients cross-recognize Sm and EBNA-1, and rabbits and mice immunized with both antigens develop SLE-like symptoms (Sabbatini et al., 1993; James et al., 1997; Poole et al., 2008). Increased numbers of EBNA-1-directed CD4 T cells are frequently found in MS and SLE patients, which often respond with an enhanced capacity to secrete IFNγ (James et al., 1994; Kang et al., 2004; Lunemann et al., 2006, 2008). Likewise, HHV-6 U24 and myelin cross-reactivity has been observed (Tejada-Simon et al., 2003; Cheng et al., 2012). Also, anti-EBNA-1 antibodies with cross reactivity against cytoskeleton proteins have been demonstrated, which could explain IAIH (Rhodes et al., 1987; Lindsey et al., 2016). A self-antibody directed against the hepatic enzyme manganese superoxide dismutase has been documented in 100% of MI patients, and mice expressing a mutant enzyme develop hepatic lesions (Ritter et al., 1994; Elchuri et al., 2005). HCMV UL94 and integrin NAG-2 cross-reactive antibodies have been found in sera of SSc patients, which induce apoptosis of NAG-2-expressing endothelial cells (Lunardi et al., 2000), and are more prominent in severe forms of SSc (Namboodiri et al., 2004). Mice immunized with HCMV result in production of common auto-antibodies present in SLE patients, and against pancreatic islet proteins, which could suggest an HCMV role in type 1 diabetes.

### Immunosenescence

Cellular senescence was first described by Hayflick et al to describe fibroblasts with a diminished capacity to proliferate *in vitro*, while remaining metabolically active for prolonged periods of time (Hayflick and Moorhead, 1961). It was subsequently shown that this phenomenon also occurs in other cells, including immune cells. Aging of the immune system is known as immunosenescence and involves a series of changes at signaling, transcriptional, epigenetic and metabolic levels, which altogether impact on the functionality of the immune system. Although immunosenescence involves all types of immune cells, here we only mention the relationship between T cell senescence and HCMV.

HCMV-induced T cell senescence is characterized by a progressive loss of naïve T cells with a concomitant increase in highly differentiated T cells. Immunosenescent T cells lack co-stimulatory receptors CD28 and CD27, and surface molecules CCR7 and CD62L. In contrast, they re-express CD45RA, KLRG1 and CD57; overall, a phenotype that resembles effector-memory T-cells. Moreover, these highly differentiated T cells (CD28^−^CD27^−^CD45RA^+^CD57^+^) are poorly proliferative, display signs of DNA damage, reduced TCR repertoire, increased production of reactive oxygen species (ROS), and progressive loss of telomeres. Of note, these cells are not anergic or dysfunctional as they show enhanced cytotoxic activity. The nutritional/metabolic status also plays an important role in T cell immunosenescence. The mTOR signaling pathway is central in effector and memory T cell development, acting jointly as a regulator of translation and as an intracellular energy sensor that regulates mitochondrial respiration (Araki et al., 2009). Highly differentiated T cells show lower mitochondrial mass and high basal glycolysis levels, which favor the use of the cytosolic glycolytic pathway over mitochondrial respiration for energy production.

About 40% of CD8 and 10% of CD4 T cells from the total memory T-cell compartment are HCMV-specific in elderly seropositive individuals (Arens et al., 2005). Interestingly, while most studies support that specific memory T cells generated by random pathogens decline with age, the frequency of HCMV-specific memory pool increases or remains stable during aging, a phenomenon termed memory T-cell inflation, of which the precise mechanism remains poorly understood (Karrer et al., 2003). Various studies suggest that these T cells are dysfunctional as defined by their senescent phenotype. In agreement, HCMV seropositive elderly individuals exhibit a highly compromised ability to respond to novel antigenic challenges, e.g., to influenza infection or vaccination. Still, other studies have shown than these cells acquire different capabilities, such as polyfunctional cytokine expression, increased cytotoxicity and antiviral reactivity (Akbar et al., 2016). Therefore, it seems that the senescence phenotype of HCMV-specific T cells do not hamper the functionality of T cells. In favor of this hypothesis, HCMV-specific T cells do not express markers of T cell exhaustion in healthy individuals either young or old, such as PD-1, TIM, LAG3, or 2B4. Altogether, these observations support that HCMV controls memory inflation and acquisition of a senescence phenotype in the aging individual. While, these T cells exhibit increased reactivity against past immune challenges (polyfunctional memory against known pathogens), they have a significantly compromised ability to respond to new threats (reduced TCR repertoire). Therefore, T cell inflation and senescence may have provided an evolutionary benefit to the population, but today, with more people living longer, it may be detrimental in old-age people. More studies are needed to completely understand this phenomenon and to find new ways to protect an increasingly aged population.

## Animal models of EBV infection to understand balanced interactions

The strict species-specific host restriction exhibited by human herpesviruses has restrained the use of animals to study viral pathogenic mechanisms and counteracting host defenses. Advances have been made with immunodeficient mice reconstituted with human hematopoietic progenitor cells. Infected humanized mice develop EBV viremia, B cell and CD8 T cell expansions, plus spleen enlargement, thus recapitulating some of the more prominent IM features. Primary infection in NK cell depleted mice results in an exacerbated form of IM with higher viremia, whopping CD8 T cell numbers that were unable to control the infection, and increased EBV tumorigenesis (Chijioke et al., 2013). A similar observation was made with CD4, CD8, or CD4/CD8 depleted mice, pointing out to the importance of T cells to control EBV infection, with CD8 T cells having a more preponderant role (Strowig et al., 2009). Indeed, transplantation of autologous EBV specific CD8 T cells induced regression of EBV positive B cell tumors (Lacerda et al., 1996). On the contrary, humanized mice infected with EBV have not been able to reproduce the viral targeting of NK or T cells observed in CAEBV. Models of NK and T cell lymphoproliferation, and of HLH have been made transplanting peripheral blood mononuclear cells (PBMC)s taken from CAEBV and HLH patients. Both CAEBV- and HLH-derived PBMCs triggered a cytokine storm in humanized mice consisting of elevated levels of IL-8, IFNγ and RANTES, with HLH-mice exhibiting greater levels of IL-8 and IFNγ than CAEBV-mice (Imadome et al., 2011). Interestingly, CD4 depletion from the CAEBV- and HLH-derived PBMCs precluded NK and T cell expansions and EBV viremia, supporting a collaborative role of CD4s in the etiology of these diseases. There is also evidence of development of RA in EBV infected humanized mice. Kuwana et al found extensive infiltration of T cells, B cells and macrophages in joints, correlating with erosive arthritis and areas of synovial cell proliferation with destruction of cartilage and bone (Kuwana et al., 2011). Two points should be carefully consider when using humanized mice to model EBV disease: (i) unlike persistent infection in humans, EBV is not found in memory B cells, and (ii) there are not self-reactive nor anti-EBV antibodies in sera of mice that could explain molecular mimicry-induced pathogenesis. Additional evidence has been generated using the murine model for experimental autoimmune encephalitis (EAE), in which an MS-like disease is induced after inoculation with myelin-derived peptides. Infection with γMHV68 exacerbates EAE (Peacock et al., 2003). Latently infected EAE mice exhibit neurological damage with an immunopathology characterized by increased CD8 infiltration, elevated levels of IFNγ and fewer Tregs, among other clinical features common to MS (Dalla Libera et al., 2011; Casiraghi et al., 2012).

## Concluding remarks

Herpesvirus long-lasting infections stem from, on one hand the virus capacity to cope with the host anti-viral immunity, and on the other, the capacity of the host to tolerate the continuous presence of the virus without appreciable self-infringed damage. A highly complex equilibrium is reached between virus and host, ultimately achieving a stage of homeostatic control in which the viral immunomodulatory capacity intimately influences on general host immune processes. This immunomodulatory capacity is significantly beneficial to the host conferring protection against multiple pathogens, autoimmune/inflammatory diseases and cancer. However, these same mechanisms responsible for boosting immune responses and protection can also bring the host to a state of disease when added genetic or environmental insults turn the balance to the pathological side. This antipodal power of herpesvirus infection would explain contradictory data in which both protective and pathogenic effects are observed.

Little is known about the genetic/epigenetic/immune elements that govern host-herpesvirus homeostasis, but as we reviewed here, acquired/iatrogenic immune disorders are teaching us of the consequences of unbalanced relationships. Although, bystander activation, disruption of tolerance and/or molecular mimicry strongly link infection with several disorders, it still does not explain how host-virus immunity is leveled, considering the rate of infected individuals vs. diseased individuals in the population. There is also a range of diseases occurring in apparently immunocompetent hosts for which we do not have a clear explanation, but that may also depend on host genetic susceptibility given by less penetrating changes than the ones responsible for PIDs, aging, co-infections, and environmental and life-style factors triggering somatic mutations that cooperate with the viral capacity for cell transformation or immunomodulation. A compelling observation is that populations with the highest and earliest frequencies of infection are the ones with the least cases of IM and AIDs. This protection could be related to the hygiene hypothesis of affluent populations, which with the introduction of sanitization products have abnormally delayed primary infection until adolescence or youth with the consequence of an increased risk for IM, AID, and some IM-associated lymphomas. In agreement, two years old children infected with EBV and HCMV had an increased number of peripheral cytokine-producing immune cells, which inversely correlated with IgE sensitization, suggesting protection against development of early atopy, allergy and asthma (Calvani et al., 1997; Nilsson et al., 2009). Interestingly, EBV-induced cancer occurs twice as often in men than in women, which could be explained by the genetic control of infection mediated by X chromosome-mapped genes. On the contrary, AIDs are more common in women than men perhaps in part because of the same reason.

The potential mutualistic nature of herpesvirus infection needs to be considered in the development of vaccines. Sterilizing immunity could override the beneficial role of these viruses, but could be valuable to control the most severe clinical symptoms observed in AIDs or PIDs. Furthermore, non-sterilizing vaccines could prevent EBV IM in seronegative teens and young adults with perhaps a concomitant reduction of IM-linked diseases such as MS and Hodgkin lymphoma. Animal models of infection should aid to test whether the vaccine does not shift the balance to other immunopathological conditions.

## Author contributions

This review was conceived and written by both corresponding authors, nurtured by their knowledge of immunodeficiencies and immunology (MC-M) and of the herpesvirus molecular biology and associated pathologies (EF-P).

### Conflict of interest statement

The authors declare that the research was conducted in the absence of any commercial or financial relationships that could be construed as a potential conflict of interest.

## Abbreviations

AID: autoimmune diseases
ALPS: autoimmune lymphoproliferative syndrome
APDS: activated PI3Kδ syndrome
CAEBV: chronic active EBV
CNS: central nervous system
CTL CD8: positive cytotoxic T lymphocytes
ds: double stranded
EAE: experimental autoimmune encephalitis
EBV: Epstein-Barr virus
FHL: familial hemophagocytic lymphohistiocytosis
HCMV/MCMV: human/murine cytomegalovirus
HHV: herpesvirus
HIV: human immunodeficiency virus
HLH: hemophagocytic lymphohistiocytosis
IAIH: idiopathic autoimmune hepatitis
IBD: inflammatory bowel disease
IFNγ: interferon gamma
IM: infectious mononucleosis
KS: Kaposi sarcoma
KSHV: Kaposi sarcoma human herpesvirus
MCD: multicentric castleman disease
MS: multiple sclerosis
PBMC: peripheral blood mononuclear cells
PEL: primary effusion lymphoma
PIDs: primary immunodeficiencies
RA: rheumatoid arthritis
SLE: systemic lupus erythematous
SSc: systemic sclerosis
TCR/BCR: T cell/B cell antigen receptor
TLR: toll like receptor
VSV: varicella zoster virus
XLP: X-linked lymphoproliferative syndrome.
